# Synthetic Associations Are Unlikely to Account for Many Common Disease Genome-Wide Association Signals

**DOI:** 10.1371/journal.pbio.1000580

**Published:** 2011-01-18

**Authors:** Carl A. Anderson, Nicole Soranzo, Eleftheria Zeggini, Jeffrey C. Barrett

**Affiliations:** Wellcome Trust Sanger Institute, Wellcome Trust Genome Campus, Hinxton, Cambridgeshire, CB10 1HH, United Kingdom; Dana-Farber Cancer Institute, United States of America

## Abstract

Synthetic associations have been posited as a possible explanation for missing heritability in complex disease. We show several lines of evidence which suggest that, while possible, these synthetic associations are not common.

The goal of human disease genetics is to connect genetic variation with disease risk, but the optimal study design for gene mapping varies widely with the underlying genetic architecture of the disease. Family based **linkage studies** made the identification of genetic defects that directly cause rare single gene diseases, like cystic fibrosis and sickle cell disease, routine. By contrast, the goal of identifying the genes that explain the heritability of complex diseases has remained elusive because they are affected by a host of genetic and environmental factors. More recently, however, genome-wide **association studies** (GWAS) have identified many common variants associated with complex traits. In some cases these studies have provided valuable insight into disease pathogenesis [1]–[3] but each associated variant often confers only a modest increase in risk (**odds ratios** [ORs] typically range from 1.1 to 1.5). One consequence of these small effects is that, even in aggregate, these discoveries fail to explain most of the heritability of complex disease. Identifying sources of this missing heritability is one of the most active areas of complex disease genetics research [4], and the relative contribution of rare (<1% minor allele frequency [MAF]), low-frequency (1%–5%), and common (>5%) causal variants remains unknown. GWAS have deeply probed the role of common variation by exploiting the fact that a subset of single nucleotide polymorphisms (SNPs) can act as proxies for (or “tag”) the majority of common SNPs. This efficiency has allowed inexpensive microarrays, which directly genotype half a million SNPs, to indirectly capture >80% of common variation [5]. While this design underpins the success of the GWAS approach, it also presents a drawback: associations to rare and low frequency SNPs are typically missed because they do not have a strongly correlated tag on GWAS chips.

Dickson et al. [6], in a recent simulation-based experiment, argue that common variant associations arising from GWAS may actually reflect multiple low frequency causal variants rather than a single common causal variant (Figure 1). They demonstrate that “synthetic associations,” where a cluster of low-frequency, highly penetrant mutations occurs stochastically more frequently with one allele than the other at a common SNP, can cause association signals at these common variants. If synthetic associations were widespread they could in theory explain an appreciable fraction of the missing heritability. Furthermore, the proportion of GWAS signals attributable to synthetic associations has profound implications for the design of GWAS follow-up studies. Therefore, while Dickson et al. argue that synthetic associations are an “obvious theoretical possibility,” it is worthwhile to broadly assess, in light of other theoretical and empirical evidence, the prevalence of synthetic associations in complex human disease.

**Figure 1:**
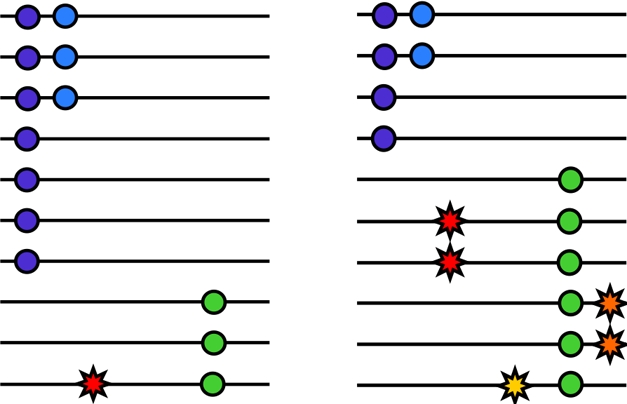
SNPs in the *NOD2* region as an example of synthetic association. Left panel represents ten control chromosomes sampled randomly; right panel represents ten random Crohn's disease patient chromosomes. Colored circles represent variant alleles at SNPs genotyped on a GWAS chip, colored explosions represent the three known causal variants in the gene. While none of the GWAS SNPs are strongly correlated with any of the individual causal alleles (the tag SNP theory which underlies the GWAS design), the collective effect of the three causal SNPs is to distort the frequencies of the GWAS SNPs in cases and controls. This collective effect of several low frequency SNPs different distances from a common SNP has been termed a “synthetic association.”

## NOD2 and Crohn's Disease: A Synthetic Association

**Figure 2:**
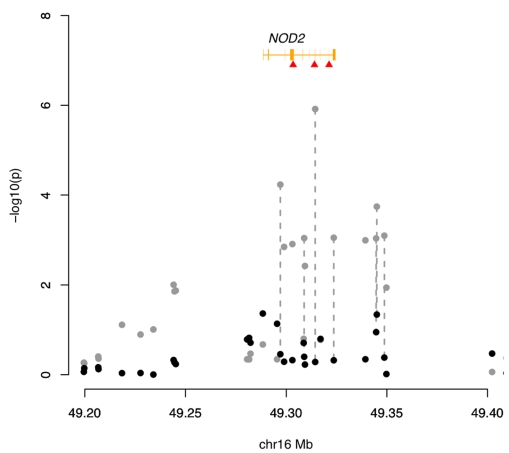
Evidence of association between Crohn's disease and the *NOD2* region. Grey points: results from a logistic regression test of association. Black points: results from a logistic regression test of association after conditioning on compound carrier status for three rare *NOD2* mutations (highlighted by red triangles). The *NOD2* gene region is denoted by the orange track. The complete eradication of the signal at the common SNPs after conditioning on the rare SNPs demonstrates that the GWAS signal is a synthetic association driven by these rare SNPs.

The synthetic association paradigm is supported [7] by the well-known association between Crohn's disease and *NOD2* [8], where three rare coding variants (G881R – MAF:0.04, R675W – MAF:0.01, and L980fs – MAF:0.02) confer high risk for Crohn's (ORs for a carrier and homozygote are 3 and 38, respectively) [9]. None of these variants are present on current GWAS arrays, nor are they individually well-tagged. Nevertheless, extremely strong association is seen at nearby common variants because the aggregate effect size of the low-frequency causal mutations is sufficiently large that it creates genome-wide significant association even at very weak, common, tag SNPs (Figure 1). For example, in the Wellcome Trust Case-Control Consortium (WTCCC) Crohn's genome-wide association study, the most strongly associated SNP in the region is rs4471699 (*P* = 1.6×10^−22^, risk allele frequency: 0.52, odds ratio: 1.52) [10]. Furthermore, in a subset of the WTCCC data where cases [11] and controls [8],[12] have been genotyped for the three low-frequency coding mutations, testing for association while conditioning on carrier status of one of these mutations completely ablates the signal at the common SNPs (minimum unconditional *P*-value = 1.2×10^−6^, conditional *P*-value = 0.52) (Figure 2). Thus, *NOD2* fulfills two important predictions of the synthetic association model: a cluster of low-frequency, high-effect variants can create a GWAS signal, and that signal vanishes when the causal alleles are taken into account.

## Linkage Versus GWAS: Power to Detect Synthetic Association

*NOD2* also illustrates an important property of the underlying genetic model proposed by Dickson et al. [6] that leads to synthetic association, namely that such loci should be amenable to linkage mapping. *NOD2* has been consistently mapped by linkage [13]–[17], in contrast to nearly all other reported linkage results for common, complex human disease. The cluster of low frequency variants at the *NOD2* locus that confer high risk for disease, which embodies the synthetic association model more generally, is highly tractable by linkage analysis. Therefore, the relative power of linkage and association can be used to make inferences about the rate at which synthetic associations occur.

**Figure 3:**
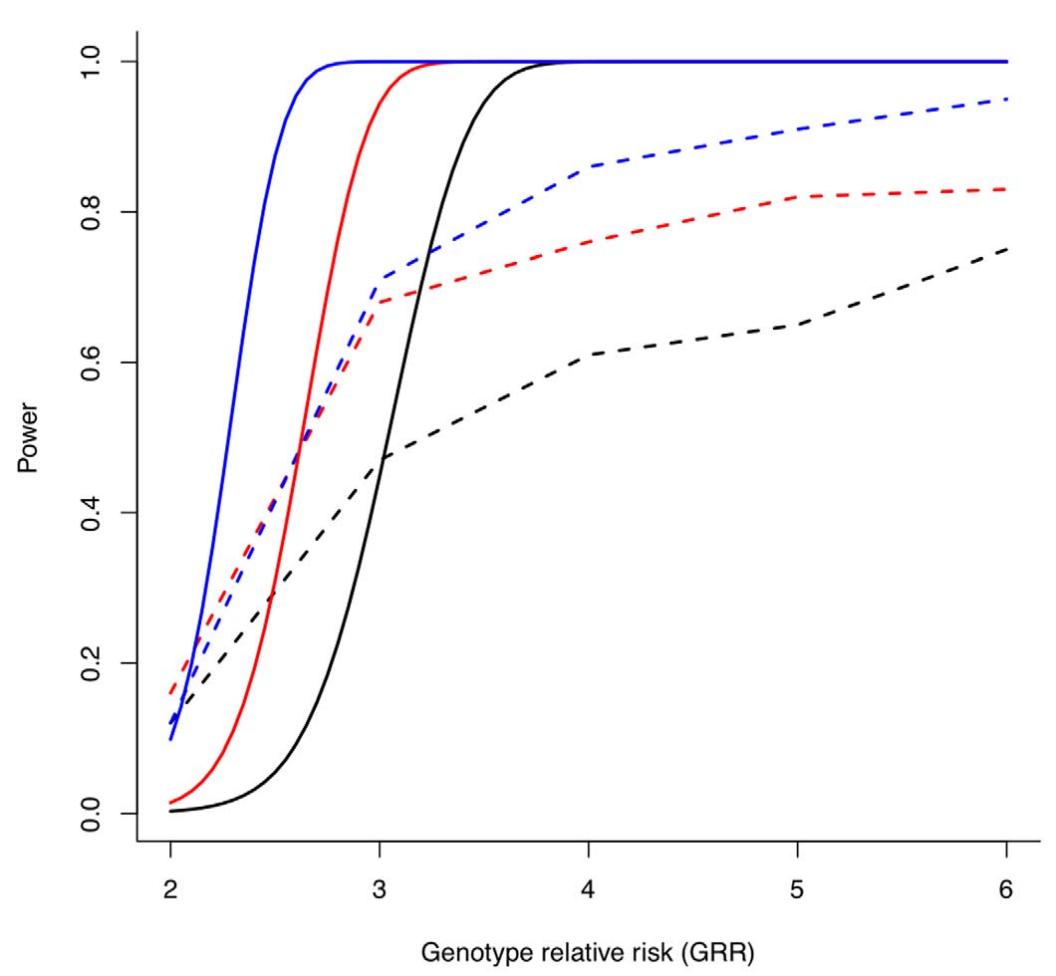
Comparative power of linkage and GWAS to detect synthetic association. Solid lines indicate power for the T1DGC linkage analysis (2,658 affected sibpairs), assuming a risk allele frequency of 0.01, and α = 1×10^−4^, for *N* = 3 (black), 5 (red), or 9 (blue) independent risk alleles. Dashed lines indicate power to detect these synthetic associations in a GWAS of 3,000 cases and 3,000 controls (data taken from Dickson et al. Figure 2). Linkage is more powerful than GWAS except for situations with few causal alleles of relatively modest effect.

Recently, the Type-1 Diabetes Genetics Consortium (T1DGC) conducted a genome-wide linkage scan of 2,658 affected sib-pairs with type-1 diabetes [18]. Using formulae outlined by Risch and Merikangas [19] it is straightforward to assess the power of this study to detect linkage given a model specifying the number of risk variants and their effect sizes. Figure 3 compares, under various models consistent with synthetic association, the power of the T1DGC linkage scan to the power of a GWAS with 2,000 cases and 2,000 controls [6]. When individual odds ratios are ≥3, or there are many independent risk variants, linkage (rather than association) is the more powerful approach. If synthetic associations are common, this observation yields a testable prediction: either GWAS signals should overlap substantially with results from well-powered linkage scans, or all synthetic associations arise from the relatively small parameter space where linkage is poorly powered but GWAS is not.

The T1DGC linkage scan shows significant linkage to the HLA region on chromosome 6p21 (LOD = 213.2), and suggestive linkage (2.2<LOD<3.6) to regions on chromosomes 2q32, 11p15, 19p13 and 19q12. Because the well-known underlying genetic effects in the HLA region (OR = 5.5) and 11p15 (*INS*, OR = 2.4) are among the strongest documented in complex disease, the linkage results at these two loci are unsurprising. The linkage signal on 2q32 could in theory be driven by synthetic associations directly under the peak (*CTLA4* (OR = 1.2) and *STAT4* (OR = 1.1) [20]), but could also be amplified by three other associations also on 2q. No documented associations (synthetic or otherwise) lie under either peak on chromosome 19. To investigate the possibility that some GWAS loci may be contained within regions displaying weak evidence of linkage (LOD>1.5), we extended the search to include an extra four such loci but did not identify any further overlaps. The remaining 47 type 1 diabetes risk loci [20] therefore show no evidence of linkage. Indeed, for complex diseases more generally there has been little overlap in the genomic regions identified using linkage and association [21]. Thus, either synthetic association is confined to a relatively narrow set of genetic models that are detectable only by GWAS or it does not account for many known associations.

## Fine-Mapping of GWAS Regions

Many groups have followed up GWAS by sequencing associated regions in large numbers of samples, with the hope of identifying common causal variants as well as additional less common, more highly penetrant mutations. The WTCCC, for instance, sequenced hundreds of samples in 16 GWAS regions (hundreds of kilobases to a megabase surrounding the most significant SNP), a design that is able to identify nearby variants causing a synthetic association. Indeed, had *NOD2* been sequenced, all three low frequency causal mutations would have been discovered. No clear examples of synthetic association were reported [22], which suggests that synthetic association is not commonplace. Nonetheless it is clear that both low- and high-frequency alleles do play a role in complex disease. Nejentsev et al. [23] recently sequenced several genes identified in a GWAS of type 1 diabetes in pools of cases, and discovered four rare coding mutations, each conferring an approximately two-fold increase in risk for diabetes. In contrast to the synthetic association model, however, these rare SNPs are not correlated with the common GWAS SNP, and a conditional analysis including these rare mutations did not affect the strength of association at the common GWAS SNP.

The dearth of synthetic associations reported to date could be due to the low-frequency variants residing outside the resequenced interval [7]. For example, analysis of a GWAS of sickle cell anemia demonstrated that strong GWAS signals can be observed at large distances from causal alleles. The genome-wide significant (*P*<5×10^−8^) signal indeed spans multiple LD blocks (2.5 Mb in total), due to the fully penetrant causal (recessive) genotype, in stark contrast to the small increase in risk from typical GWAS hits. Even so, the most associated SNP (*P* = 1.1×10^−136^) is within 10 kb of the known functional variant in *HBB* [6]. The notion that GWAS signals are typically located close to underlying functional elements is further supported by their frequent proximity to candidate genes associated with related Mendelian conditions or identified by pathway analyses [24],[25]. For example, eight of ten proteins involved in the Th17-differentiation signaling pathway have been associated with one or more auto-inflammatory diseases [26]. Identifying so many candidate genes within the narrow intervals around GWAS signals suggests that causal variants are not routinely located megabases away from the most strongly associated common SNP.

While these studies are not exhaustive, they provide three insights into the functional architecture of complex disease. First, both low-and high-frequency risk alleles can independently exist within a single locus. Second, GWAS signals often map closely to underlying functional elements. Finally, the more general lack of variants with obvious functional consequences that would explain the association in GWAS regions suggests that sequencing of larger sample sets, as well as better functional annotation of regulatory elements, will be required.

## Population Genetics of Synthetic Associations

In contrast to common SNPs, most low-frequency SNPs are not shared across diverged populations because they have either arisen relatively recently or their frequencies have been influenced by population history (e.g., the out-of-Africa expansion or natural selection). Since the synthetic associations proposed by Dickson et al. are created by low-frequency variants, they are therefore less likely to be shared among diverged populations. The Crohn's disease synthetic association at *NOD2* is again illustrative, as it is restricted to populations of European or Jewish descent [27]–[29]. The three rare causal mutations are not observed in Asian populations, so the common variant (which is observed at similar frequency to that seen in individuals of European descent) is not associated with Crohn's disease in individuals of Asian descent. In contrast, many disease loci initially identified via GWAS have been widely replicated across divergent populations, supporting the hypothesis that the causal mutations are, in fact, common [30]–[32]. Some population-specific GWAS signals do exist [6], but caution is required in the interpretation of small-scale, population-specific replication studies of loci originally identified in meta-analyses involving thousands of samples. Larger sample sizes from populations around the world are needed to resolve the fraction of GWAS hits that are population-specific.

## Conclusions

Complex human diseases are influenced by common, low-frequency, and rare mutations, and a hypothesis invoking multiple rare variants (as proposed by Dickson) is compatible with the common disease common variant hypothesis [4]. Large-scale sequencing studies of thousands of cases and controls will be required to fully understand the genetic architecture of complex disease. Since the prevalence of synthetic association acutely affects the design of these studies (i.e., in terms of the sample composition and width of genomic region sequenced) it is worth carefully evaluating its contribution to missing heritability. The balance of current evidence suggests that this contribution is likely to be small. Linkage results, preliminary evidence from targeted sequencing studies, pathway analyses and transcontinental replication indicate that common SNP associations arising from GWAS are just that: common SNP associations.

**Box 1. Glossary**

**Odds ratio:** the ratio of odds of disease between two groups that differ for a variable of interest. In genetics, the variable is genotype at a particular location in the genome; therefore the odds ratio is a measurement of the effect size of that genetic variant on disease risk. Single-gene disorders, where a particular mutation essentially guarantees disease, have infinitely large odds ratios, whereas common risk variants for complex diseases typically have odds ratios around 1.1–1.5.

**Linkage study:** a gene-mapping approach that searches for genomic segments that are co-inherited with disease within a family. Linkage scans have been most successful in the study of diseases caused by rare mutations in a single gene.

**Association study:** another gene-mapping study design based on comparing allele frequencies between cases and controls. Inexpensive genotyping technologies coupled with large patient collections (thousands of individuals with a disease) have enabled genome-wide association studies that are well suited to detecting small effects at common SNPs.
